# Bioenergetic profile of human coronary artery smooth muscle cells and effect of metabolic intervention

**DOI:** 10.1371/journal.pone.0177951

**Published:** 2017-05-19

**Authors:** Mingming Yang, Amy E. Chadwick, Caroline Dart, Tomoko Kamishima, John M. Quayle

**Affiliations:** 1 Department of Cellular and Molecular Physiology, University of Liverpool, Liverpool, United Kingdom; 2 Department of Molecular and Clinical Pharmacology, University of Liverpool, Liverpool, United Kingdom; 3 Department of Biochemistry, University of Liverpool, Liverpool, United Kingdom; University of Nebraska Medical Center, UNITED STATES

## Abstract

Bioenergetics of artery smooth muscle cells is critical in cardiovascular health and disease. An acute rise in metabolic demand causes vasodilation in systemic circulation while a chronic shift in bioenergetic profile may lead to vascular diseases. A decrease in intracellular ATP level may trigger physiological responses while dedifferentiation of contractile smooth muscle cells to a proliferative and migratory phenotype is often observed during pathological processes. Although it is now possible to dissect multiple building blocks of bioenergetic components quantitatively, detailed cellular bioenergetics of artery smooth muscle cells is still largely unknown. Thus, we profiled cellular bioenergetics of human coronary artery smooth muscle cells and effects of metabolic intervention. Mitochondria and glycolysis stress tests utilizing Seahorse technology revealed that mitochondrial oxidative phosphorylation accounted for 54.5% of ATP production at rest with the remaining 45.5% due to glycolysis. Stress tests also showed that oxidative phosphorylation and glycolysis can increase to a maximum of 3.5 fold and 1.25 fold, respectively, indicating that the former has a high reserve capacity. Analysis of bioenergetic profile indicated that aging cells have lower resting oxidative phosphorylation and reduced reserve capacity. Intracellular ATP level of a single cell was estimated to be over 1.1 mM. Application of metabolic modulators caused significant changes in mitochondria membrane potential, intracellular ATP level and ATP:ADP ratio. The detailed breakdown of cellular bioenergetics showed that proliferating human coronary artery smooth muscle cells rely more or less equally on oxidative phosphorylation and glycolysis at rest. These cells have high respiratory reserve capacity and low glycolysis reserve capacity. Metabolic intervention influences both intracellular ATP concentration and ATP:ADP ratio, where subtler changes may be detected by the latter.

## Introduction

The energy storing molecule ATP fuels a variety of cell functions including maintenance of transmembrane ionic gradients, muscle contraction, secretion, cell proliferation and migration. It also acts as an intracellular signaling molecule that translates cellular metabolic status to physiological responses. ATP is also required for phosphorylation, a central step in a myriad of signal transduction cascades involving kinases. Often taken for granted, bioenergetics underpins all forms of life, but its importance is revealed when its dysfunction results into serious diseases such as diabetes mellitus, inherited mitochondrial disorders, metabolic syndrome and neurodegeneration [1].

For multicellular organisms, the traditional view is that oxygen-consuming mitochondrial oxidative phosphorylation (OXPHOS) is the preferred ATP production route due to its superior efficiency. Indeed, ATP production by anaerobic glycolysis is thought to be inhibited when ATP production rate by OXPHOS is high (the Pasteur effect) [2]. Cancer cells, however, are known to favor glycolysis even when they are well oxygenated. This is aerobic glycolysis, also known as the Warburg effect [2]. Recently, however, the notion that the Warburg effect is unique to cancer cells has been challenged as aerobic glycolysis is seen among non-cancerous proliferating cells including vascular smooth muscle cells [3]. This may have an intriguing implication in vascular diseases such as atherosclerosis. Unusual amongst terminally differentiated cells, vascular smooth muscle cells have the ability to dedifferentiate and switch from a contractile to a proliferating phenotype [4]. Though essential in repairing vascular injury, dedifferentiation is the key step at the onset of atherosclerosis where proliferating and migrating vascular smooth muscle cells initiate cap formation [4].

The relative contribution of OXPHOS and glycolysis to ATP production determines the macroscopic bioenergetic profile, which may shift according to changes in cellular phenotype or metabolic status. In addition, determination of OXPHOS and glycolysis reserve capacities may be a useful indicator of cell resilience in time of emergency. Reserve capacity is particularly important in high energy-consuming cardiovascular and neuronal systems where the failure to supply adequate amounts of ATP could quickly lead to catastrophic events. Despite its significance, however, bioenergetics of non-cancer cells is not widely characterized. The lack of information in cell bioenergetics arises, at least in part, from the difficulty in determining OXPHOS and glycolysis simultaneously from homogeneous intact cell populations [5, 6]. Historically, isolated mitochondria were used for examination of bioenergetics, but isolated mitochondria may behave quite differently from those within intact cells [7]. Attempts were made previously to determine bioenergetic profile using tissues [8]. However, tissues are composites of heterogeneous cell populations, so experiments meant to examine artery smooth muscle cells were carried out in the presence of other type of cells including endothelial cells. Although it was necessary to use tissues due to available detection methods, it is now possible to measure OXPHOS and glycolysis from a defined cell population. The Seahorse technique is a recently developed method that simultaneously monitors the cellular oxygen consumption rate (OCR) and the extracellular acidification rate (ECAR) [7, 9]. The former is a measurement of the aerobic component while the latter is an indicator of lactate production and thus the glycolytic component. Detection is highly sensitive, allowing measurements from a relatively small number of cells. To date, however, the Seahorse technique has not been widely exploited outside of cancer research.

We sought to examine cellular bioenergetic profile of cultured human coronary artery smooth muscle cells (HCASMCs). Coronary arteries are particularly susceptible to atherosclerosis that can lead to myocardial infarction [4]. Understanding cellular bioenergetics in proliferating smooth muscle cells may be useful for identifying possible cellular targets in rogue proliferating cells while sparing normal, non-proliferating smooth muscle cells [6]. Also, homeostasis of nucleotides is important in coronary artery smooth muscle cells. One of the consequences of increased metabolic demand is that cells utilize more ATP, and this initial mis-match in demand and supply is dealt with by increased coronary blood flow. One of the mechanisms proposed, but not completely deciphered, is metabolic activation of ATP sensitive K^+^ (K_ATP_) channels [10] where a decrease in ATP and increase in ADP level is thought to hyperpolarize coronary artery smooth muscle cells. K_ATP_ channels are largely closed at rest when the ATP level is high and the ADP level is low. When cellular metabolic demand increases, however, tonic inhibition of K_ATP_ channels is removed due to decrease in ATP level with a concomitant increase in the ADP concentration. K^+^ efflux through K_ATP_ channels hyperpolarizes the cell membrane, leading to the closure of voltage-dependent Ca^2+^ channels, a decrease in intracellular Ca^2+^ concentration and muscle relaxation and coronary artery dilation [11, 12]. Thus, ATP and ADP in HCASMCs may act as intracellular signaling molecules, linking cellular metabolism to increased blood flow. Thus, we also sought to exploit the recent development in biosensors that detect ATP:ADP ratio in living cells. Perceval and its improved version, PercevalHR, are fluorescent biosensors of adenylate nucleotides [13, 14]. ATP and ADP compete for the same binding site of Perceval and PercevalHR with high affinity. Binding of ADP shifts the chromophore charge state equilibrium, favoring the neutral protonated state while binding of ATP shifts to the anionic deprotonated state. Together, Perceval and PercevalHR are thought to act as biosensors of intracellular ATP:ADP ratio [13, 14 but see 15].

In the current study, the bioenergetic profile of HCASMCs and the effects of metabolic intervention were examined. Real-time respirometry, using Seahorse technology, was performed to dissect out defined components of OXPHOS and glycolysis. The change in bioenergetic profile caused by metabolic modulators and the aging of cells during culture was also evaluated. Effects of metabolic modulators on ATP level, mitochondrial membrane potential and intracellular ATP:ADP ratio were also evaluated. These techniques have enabled us to report quantitative analysis of cellular bioenergetics of HCASMCs.

## Materials and methods

### HCASMCs, reagents and equipment

Cryopreserved primary HCASMCs (cat # C-12511), obtained from Caucasian males aged 54 and 34, were purchased from Promocell (Heidelberg, Germany) at passage 2 and cultured in Promocell smooth muscle cell growth medium 2 supplemented with Promocell smooth muscle cell growth medium 2 supplement mix (cat # C-22062). Each set of experiments were performed using cells originating from the same donor. Consumables for Seahorse experiments were purchased from Seahorse Bioscience. CellTiter-Glo luminescent cell viability assay kit (cat # G7572) was purchased from Promega. Oligomycin was purchased from Abcam Biochemicals (cat # AB143424), and carbonyl cyanide *p*-trifluoromethoxyphenylhydrazone (FCCP, cat # C2920), rotenone (cat # R8875), antimycin (cat # A8674), 2-deoxy-D-glucose (2-DG, cat # D6134), carbonyl cyanide *m*-chlorophenyl hydrazone (CCCP, cat # C-2759) were purchased from Sigma-Aldrich. Rhodamine123 was purchased from Molecular Probes (cat # R302). Plasmid maxi kit was bought from Qiagen (cat # 12662). All other chemicals were from Sigma-Aldrich or Fisher Scientific. GW1-PercevalHR and FUGW-PercevalHR were gifts from Gary Yellen (Addgene plasmids # 49082 and 49083) [14], and all other materials required for lentivirus experiments were kind gifts from Dr. Joanna Wardyn (University of Liverpool). The Seahorse XF^e^96 Analyser (Seahorse Bioscience) was used to simultaneously monitor aerobic and glycolytic components of cellular bioenergetics. A plate reader (FLUOstar Omega, BMG LABTECH) was used for luminescence and fluorescence recordings. Fluorescence signal was collected with LSM510 multiphoton confocal microscopy that allowed control of CO_2_, humidity and temperature (Carl Zeiss AG, Oberkochen, Germany), and data were analyzed using AIM software (version 3.2 SP2).

### Seahorse stress tests

Seahorse technology utilizes fluorescent probes sensitive to oxygen and protons to determine OCR and ECAR, respectively. In practice, the latter is the measurement of acidification of the extracellular solution caused by lactate production and CO_2_ generation. Thus, all measurements were carried out using an unbuffered XF DMEM (cat # 102353–100). HCASMCs were plated into Seahorse 96-well XF cell culture microplates (cat # 101085–004) at a density of 2.0x10^4^ cells per well using fully supplemented media and left overnight. To prime the sensor cartridge, 200 μl of XF calibrant solution (cat # 100840–000) was added to each well of XF^e^96 FluxPaks (cat # 102416–100), and the sensor cartridge was submerged and left overnight at 37°C in a CO_2_ free incubator to hydrate. The following day, the media was replaced with unbuffered DMEM containing 1 mM sodium pyruvate, 25 mM D-glucose and 2 mM L-glutamine for mitochondria stress test. Media was replaced with unbuffered glucose free DMEM for glycolysis stress test. The cell microplate with sensor cartridge was then incubated at 37°C in a CO_2_ free environment for a minimum of 1 hr before stress tests. Readings were normalized against total protein content of each well. Thus, at the end of the assay, solution in the wells was removed, and 20 μl of somatic cell ATP releasing reagent (Sigma, cat # FLSAR-1VL) was added and vortexed for 5 minutes to lyse the cells. The total protein in each well was determined by BCA assay. OCR and ECAR rates were expressed against either per μg protein or per 2.0x10^4^ cells.

### CellTiter Glo luminescent cell viability assay

To measure ATP levels, 100 μl of the cell suspension containing 2,500 cells was plated into a 96-well flat bottom black polystyrene plates and left in a 37°C/5%CO_2_ incubator for 24–48 hours. Intracellular ATP was released by addition of 100 μl CellTiter-Glo reagent to lyse the cells. The plate was incubated for 2 min on an orbital shaker, with further 10 min incubation at room temperature to stabilize the signal. Luminescence signal was recorded using a plate reader and expressed as relative luminescent units (RLU).

### HCASMC volume estimation

In order to determine cell volume, HCASMCs expressing membrane targeted green fluorescent probe were lifted from culture dishes with trypsin and suspended in cell culture medium. Images of spherical cells were taken using a LSM510 multiphoton confocal microscope and analyzed to determine the major axis and the minor axis of best fitting ellipse using Fiji software.

### Mitochondrial membrane potential measurement

To measure the change in mitochondrial membrane potential (ψ_m_), HCASMCs were plated into 35 mm glass-bottom dishes. On the day of experiments, cells were washed twice with bicarbonate buffered physiological saline solution (PSS) containing [mM]: 120 NaCl, 5 KCl, 1 MgCl_2_, 2 CaCl_2_, 0.42 Na_2_HPO_4_, 0.44 NaH_2_PO_4_, 24 NaHCO_3_ and 10 glucose. Cells were incubated in PSS containing 10 μg/ml rhodamine123 for 15 min in a 37°C/5%CO_2_ incubator, and dye loading was stopped by washing cells with PSS twice. Rhodamine123 was excited at 514 nm, and emission was collected at 520–555 nm. In order to minimize signals originating from plasma membrane [16, 17], extracellular K^+^ was raised to 60 mM by replacing equimolar Na^+^ during the course of rhodamine123 measurements.

### ATP:ADP ratio microplate assay

To generate HCASMCs expressing FUGW-PercevalHR, a conventional 3^rd^ generation HIV-based lentivirus packaging system was used [18]. The ampicillin resistant plasmids (RRE, REV, VSVG) were used to transform competent *E*.Coli cells. Transformation of *E*.Coli, growth of transformed cells and plasmid isolation were performed following the manufacturer’s protocol. Successful transformation was confirmed by restriction analysis. The purified plasmids were prepared with maxi-prep, then transiently transfected into the packaging cell line HEK293T cells, either by Ca^2+^ precipitation or by using commercially available transfection reagents. The resulting lenti-pseudoviral particles were secreted into culture media and collected. Lenti-pseudoviral particles were frozen and stored in a -80°C freezer or used immediately to infect HCASMCs [19]. The vector was added and incubated with the cells for 16–24 hrs, then the media was completely removed, and fresh media was added. The infected cells express the fluorescent reporter protein as a result of viral integration into the host genome. Cells expressing FUGW-PercevalHR were seeded into a 96-well black polystyrene plate at a density of 2,500 cells per well. Fluorescence was recorded every 1 min with a plate reader with the excitation of 485 nm and emission of 520 nm.

### Statistical analysis

When appropriate, values are expressed as a mean±standard error of mean (SEM). n indicates number of cells, wells or plates unless otherwise stated. Statistical significance was evaluated with a Student’s *t*-test or with ANOVA with Tukey’s test for post hoc analysis using SPSS software package (version 20.0, IBM).

## Results

### Defining the bioenergetic phenotype of HCASMCs

**Fig 1:**
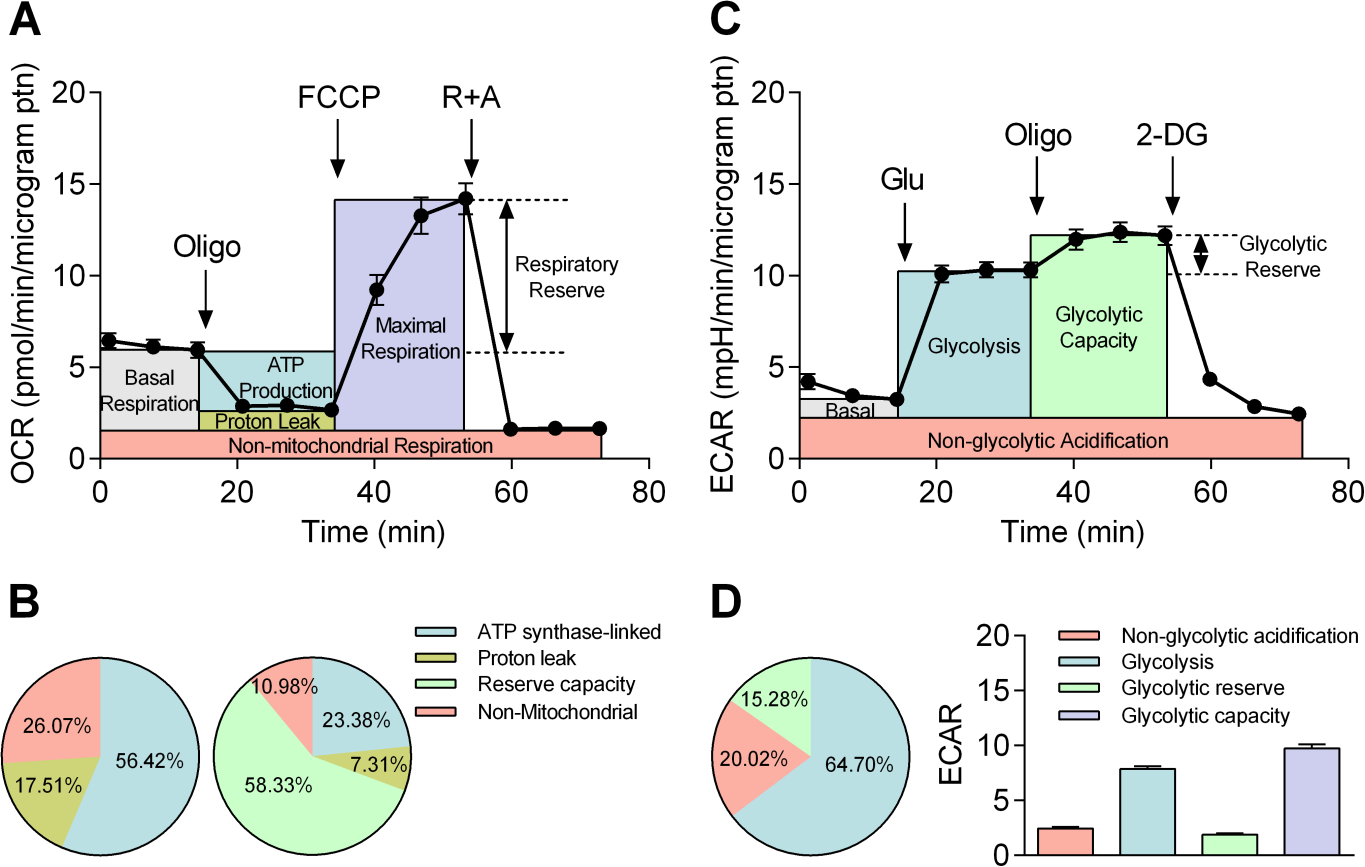
Bioenergetic phenotyping using Seahorse technology. A: Mitochondrial stress test where OCR is expressed per μg protein. Indices of mitochondrial function, basal respiration, ATP production, proton leak, maximal respiration, respiratory reserve and non-mitochondrial respiration, were compartmentalized by a sequential application of pharmacological inhibitors, oligomycin, FCCP and a combination of rotenone and antimycin (R+A). B: The proportion of OCR due to ATP synthase, proton leak and non-mitochondrial oxygen consumption was quantified by taking “resting” OCR as 100% (left panel). The proportion of above three indices plus reserve capacity is quantified by taking “highest” OCR as 100% (right panel, n = 8). Note that “resting” and “highest” OCR are the sum of basal/maximal OCR and non-mitochondrial OCR. C: Glycolysis stress test was carried out by a sequential application of glucose (Glu) pharmacological inhibitors of OXPHOS (Oligo) and glycolysis (2-DG) to dissect basal acidification, glycolysis, glycolytic capacity, glycolytic reserve and non-glycolytic acidification. Change in ECAR is expressed per μg protein. D: ECAR is shown either as percentage of three components (glycolysis, glycolytic reserve, and non-glycolytic acidification) by taking “highest” ECAR as 100% (left panel) or per μg protein including glycolytic capacity, a sum of glycolysis and glycolytic reserve (right panel, n = 8).

The Seahorse technique can be used to quantify OXPHOS and glycolysis by using specific protocols designed to dissect distinct components with pharmacological agents. Thus, mitochondrial stress tests and glycolysis stress tests were carried out to investigate OXPHOS and glycolysis, respectively (Fig 1). In both stress tests, the Seahorse technology generates the time course of OCR and ECAR. Using the mitochondrial stress tests, the “resting” OCR was first established, and cells were treated with 1.0 μM oligomycin (Fig 1A). Oligomycin is an inhibitor of ATP synthase, and so the difference before and after oligomycin application isolates the OCR linked to ATP production. Subsequent application of a protonophore, FCCP (0.75 μM), provides the reading of the “highest” OCR. Reserve capacity can therefore be calculated as the difference between “resting” and “highest” OCR. Finally, rotenone and antimycin (both 1 μM) were applied to block complex I and complex III to fully inhibit the electron transport chain (ETC). Thus, residual OCR in the presence of rotenone and antimycin can be attributed to non-mitochondrial oxidases [1]. From this protocol, three further parameters were calculated. First, maximal OCR can be calculated as the difference between “highest” OCR and non-mitochondrial OCR. Second, proton leak can be calculated as the difference between OCR in the presence of oligomycin and OCR in the presence of rotenone and antimycin. Third, the true basal OCR can be determined as the difference between “resting” OCR and non-mitochondrial OCR. To summarize, the proportion of OXPHOS components is expressed by either taking “resting” OCR (Fig 1B left panel) or “highest” OCR (Fig 1B right panel) as 100% (n = 8). The optimal concentrations of oligomycin and FCCP were titrated prior to the mitochondrial stress test, and results are shown in supporting information (S1 Fig).

Next, the glycolysis stress tests were performed to determine glycolytic activity in HCASMCs by determining ECAR. In practice, Seahorse technology measures proton production rate (PPR) from which ECAR can be calculated. First, 10 mM glucose was added to the cells kept in glucose free unbuffered DMEM (Fig 1C). Next, oligomycin was applied to reveal the “highest” ECAR by halting mitochondrial ATP production. Finally, ECAR due to non-glycolytic acidification was measured by applying 2-DG as a pseudosubstrate. The 2-DG resistant, non-glycolytic component is likely to arise from other acidification such as CO_2_ production by cells. In addition to non-glycolytic ECAR, the glycolysis stress test generates four additional indices: First, glycolytic capacity can be calculated as the difference between “highest” ECAR and non-glycolytic acidification. Second, glycolysis can be calculated as the difference between ECAR in the presence of glucose and 2-DG. Third, glycolytic reserve can be determined as the difference between “highest” ECAR and ECAR in the presence of glucose. Fourth, the true basal ECAR can be determined as the difference between “resting” ECAR and non-glycolytic ECAR. Fig 1D shows the proportion of glycolysis, glycolytic reserve and non-glycolytic ECAR by taking “highest” ECAR as 100% (left panel) with actual values of each component plus that of glycolytic capacity (sum of glycolysis and glycolytic reserve) (right panel).

**Fig 2:**
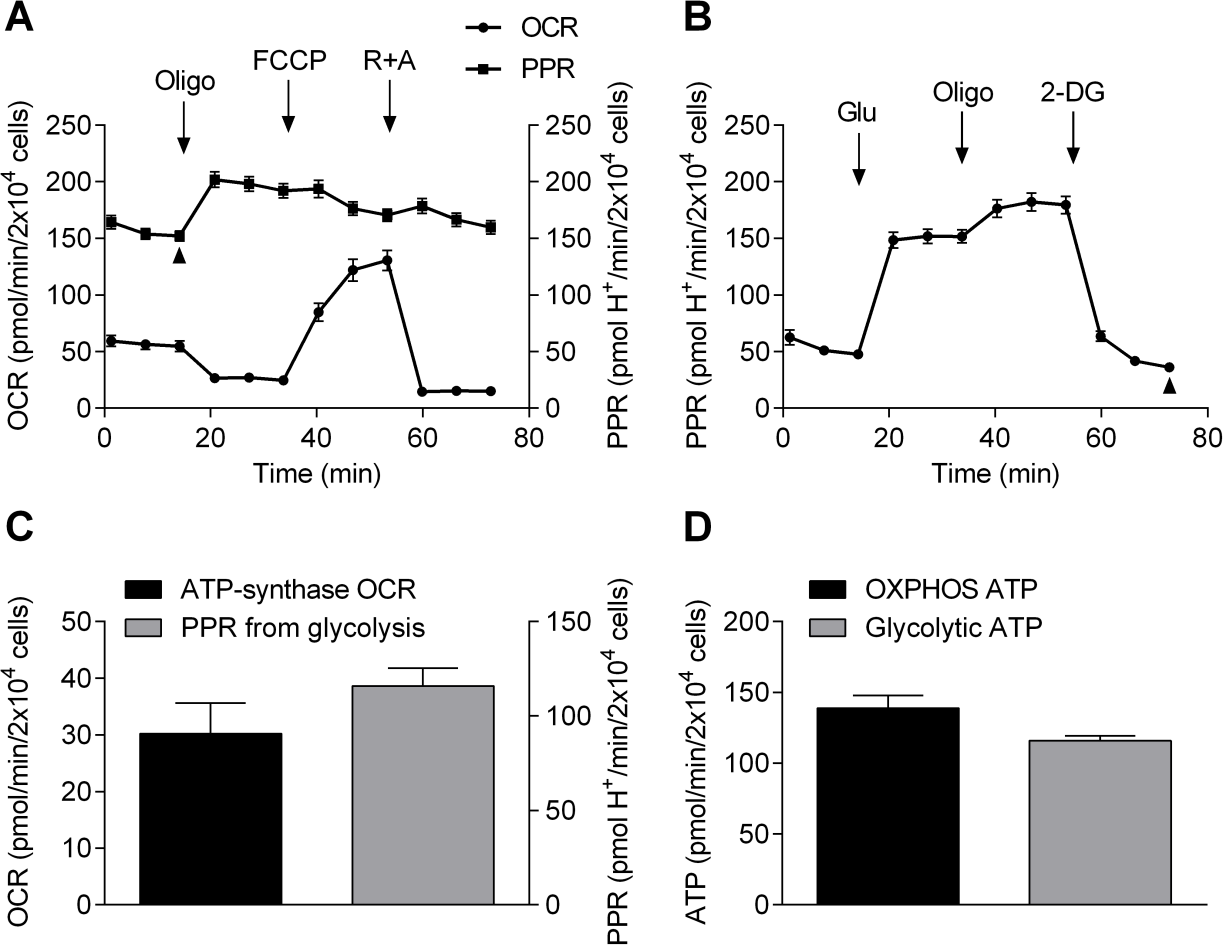
ATP production rate from OXPHOS and glycolysis. A: OCR (circle, left Y axis) and PPR (square, right Y axis) of mitochondrial stress test, both expressed as per 2.0x10^4^ cells. R is rotenone and A is antimycin B: PPR of glycolysis stress test expressed as per 2.0x10^4^ cells. C: OCR from OXPHOS (left bar, left Y axis) and PPR from glycolysis (right bar, right Y axis) expressed as per 2.0x10^4^ cells (n = 8). D: ATP production rate from OXPHOS (left bar) and glycolysis (right bar) expressed as per 2.0x10^4^ cells (n = 8, p = 0.03).

To estimate the proportion of ATP generated by OXPHOS and glycolysis in HCASMCs, we further analyzed the results from the stress tests. Fig 2A shows the PPR determined during mitochondrial stress tests, along with OCR shown previously in Fig 1A. PPR from glycolysis stress tests is shown in Fig 2B. As described above, oligomycin dissects out OCR caused by ATP production (left column, Fig 2C). Arrow heads show “resting” PPR during mitochondrial stress test (Fig 2A) and non-glycolytic PPR in the presence of oligomycin and 2-DG during glycolysis stress test (Fig 2B). Non-glycolytic PPR accounted for 23.74% of the “resting” PPR (36.00/151.64 pmol H^+^/min per 2x10^4^ cells), and the difference is due to glycolysis (right column, Fig 2C). The values shown in Fig 2C can be converted to ATP production rates. First, ATP production from OXPHOS was calculated from oligomycin-sensitive OCR (30.20 ± 1.93 pmol/min per 2x10^4^ cells) using a phosphate/oxygen ratio of 2.31 [20]. PPR from glycolysis does not require calculation as glycolysis generates two H^+^ and two ATP, making the relationship between PPR and ATP production 1:1. Thus, HCASMCs cultured in high glucose DMEM containing 1 mM sodium pyruvate and 2 mM L-glutamate generate ATP at a rate of 138.93±8.88 pmol/min per 2x10^4^ cells from OXPHOS and 115.87±3.33 pmol/min per 2x10^4^ cells from glycolysis (Fig 2D). These results indicate that HCASMCs produce 54.5% of ATP through OXPHOS at rest with the remaining 45.5% due to glycolysis.

### Bioenergetic phenotype shift caused by application of metabolic modulators

**Fig 3:**
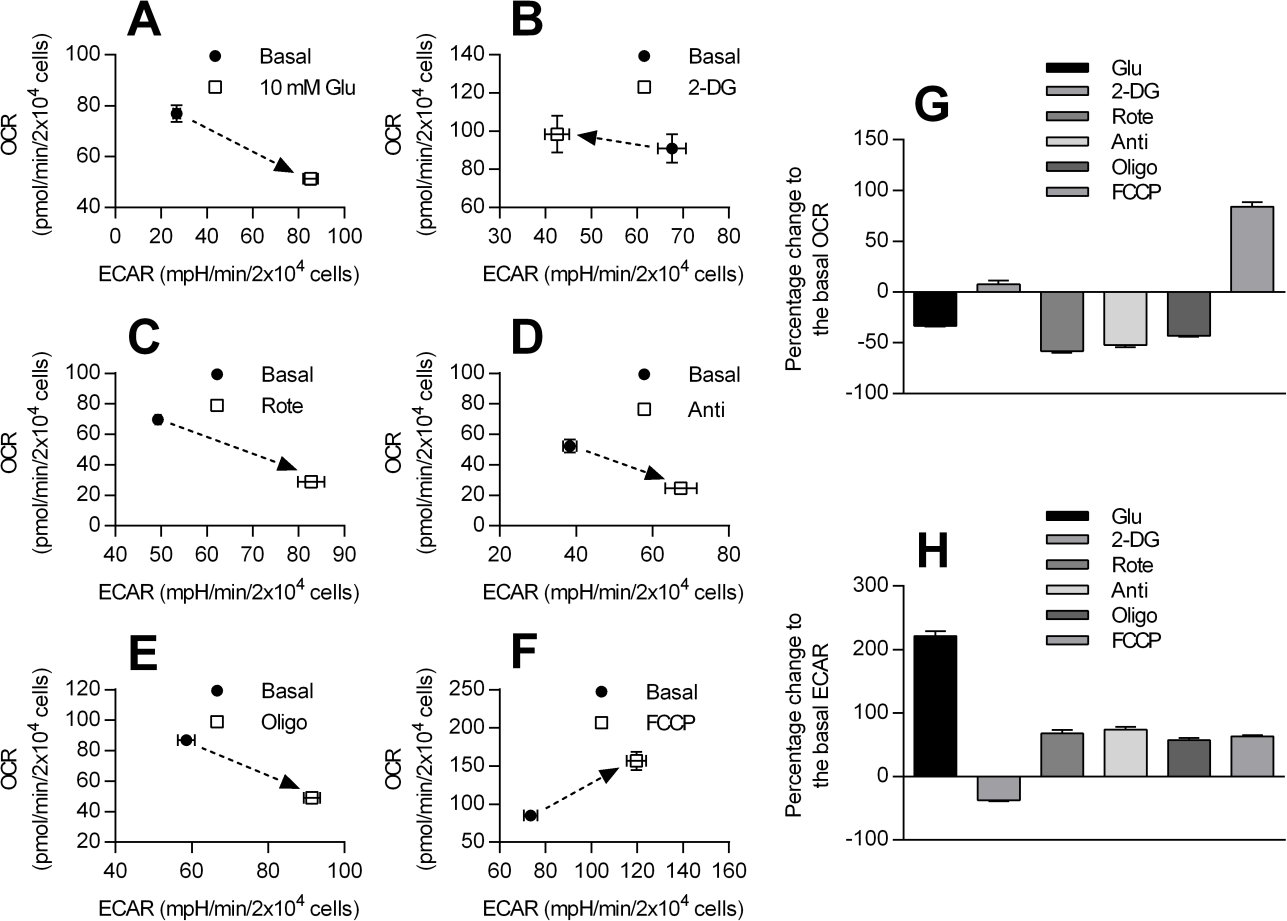
Shift in bioenergetic phenotype in response to metabolic modulators. The change in relative bioenergetic phenotype of HCASMCs after exposure to 10 mM glucose (in glucose-free medium) (A, n = 8), 5 mM 2-DG (B, n = 8), 1 μM rotenone (C, n = 8), 1 μM antimycin (D, n = 7), 1 μM oligomycin (E, n = 8) and 0.75 μM FCCP (F, n = 8). Shift in bioenergetic phenotype can be detected as relative positional change from basal value (filled circle) after drug application (open square) when ECAR is plotted on the X axis and OCR on the Y axis. Panel G and H summarize percent changes of OCR and ECAR against control level.

Next, the effect of metabolic modulators on bioenergetic profile of HCASMCs was analyzed. When OCR is plotted as a function of ECAR before and after pharmacological intervention, the plot can be partitioned into four characteristic subsets: low ECAR/low OCR as quiescent, high ECAR/low OCR as glycolytic, low ECAR/high OCR as aerobic and high ECAR/high OCR as energetic. Thus, the relative positional change caused by metabolic modulators reflects the shift in bioenergetic phenotype. Addition of glucose to glucose-free DMEM caused a shift from aerobic to glycolytic (Fig 3A). 2-DG shifted the bioenergetic phenotype to be more aerobic (Fig 3B). Rotenone, antimycin and oligomycin caused the cells to become more glycolytic (Fig 3C, 3D & 3E). FCCP shifted the relative HCASMC bioenergetic phenotype from quiescent to energetic (Fig 3F). Percentage change in OCR and ECAR against basal level in response to metabolic modulators is summarized in Fig 3G and 3H.

### Bioenergetic phenotype shift caused by cell age

**Fig 4:**
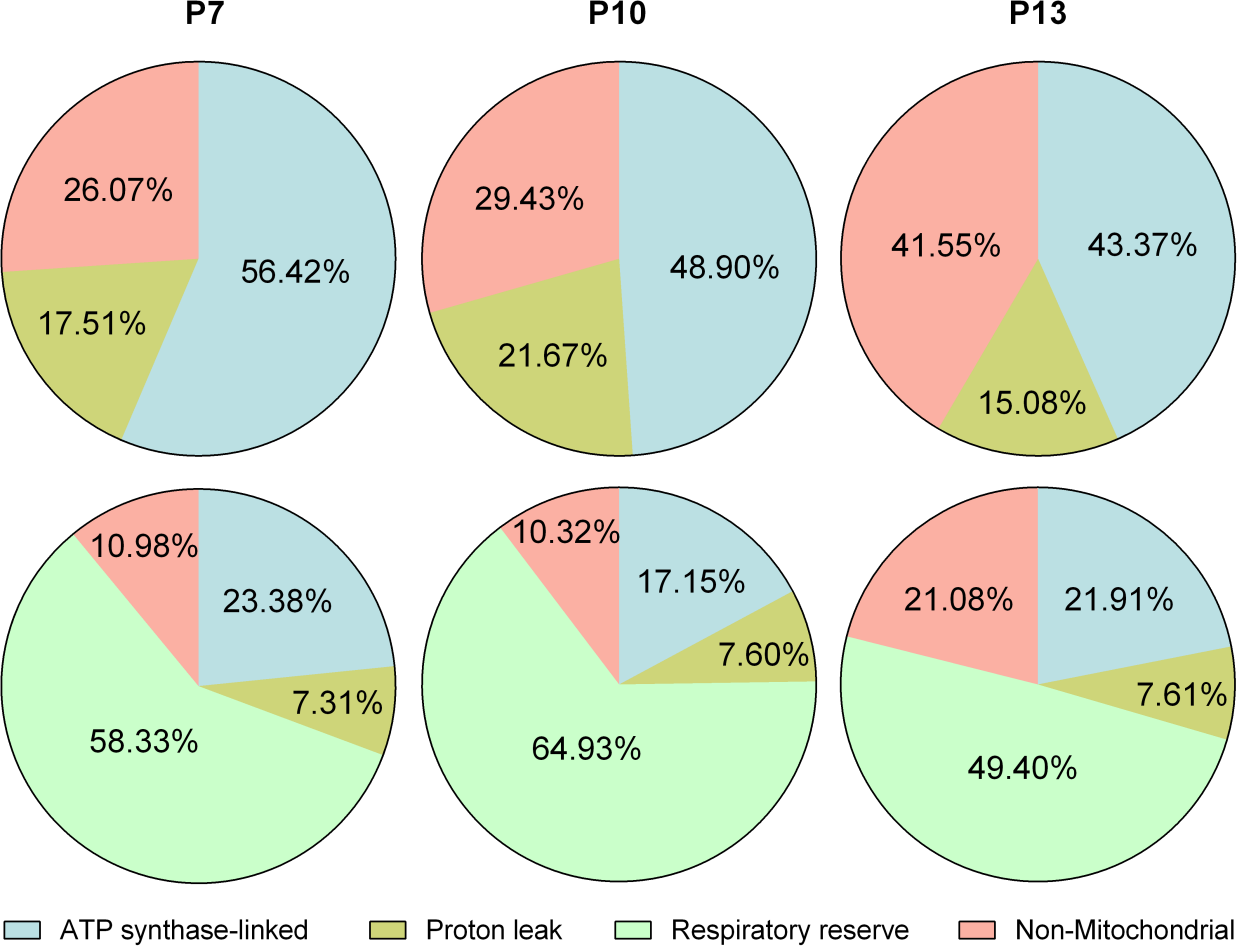
Cellular bioenergetics of HCASMCs during culture. Summary of mitochondrial stress test using cells at passage (P) 7, 10 and 13. The proportion of OCR due to ATP synthase, proton leak and non-mitochondrial oxygen consumption when “resting” OCR was taken as 100% (top panels). The proportion of above three indices plus reserve capacity when “highest” OCR was taken as 100% (bottom panels, n = 8). Note that results of P7 are previously shown in Fig 1B.

As described earlier, the cellular bioenergetic profile may shift during both physiological and pathological events. It is also known that the phenotype of primary cells changes with time during cell culture. Thus, the OXPHOS related bioenergetics profile was compared among passage 7, 10 and 13 HCASMCs. During passage, there was little change in the expression of smooth muscle signature proteins including α-actin, calponin, and myosin heavy chain determined by Western blot and immunocytochemistry (data not shown). Immunocytochemistry results showed that virtually all cells express smooth muscle signature proteins, and therefore any change in bioenergetics is not due to increase in the proportion of non-smooth muscle cells. Fig 4 shows that as the number of passages increased, the proportion of ATP synthase-linked OCR and reserve capacity decreased. Non-mitochondrial OCR, on the other hand, increased with passage number. These results suggest that cell senescence is associated with lower OXPHOS, both at rest and in reserve.

### Effects of metabolic inhibitors on cellular ATP

**Fig 5:**
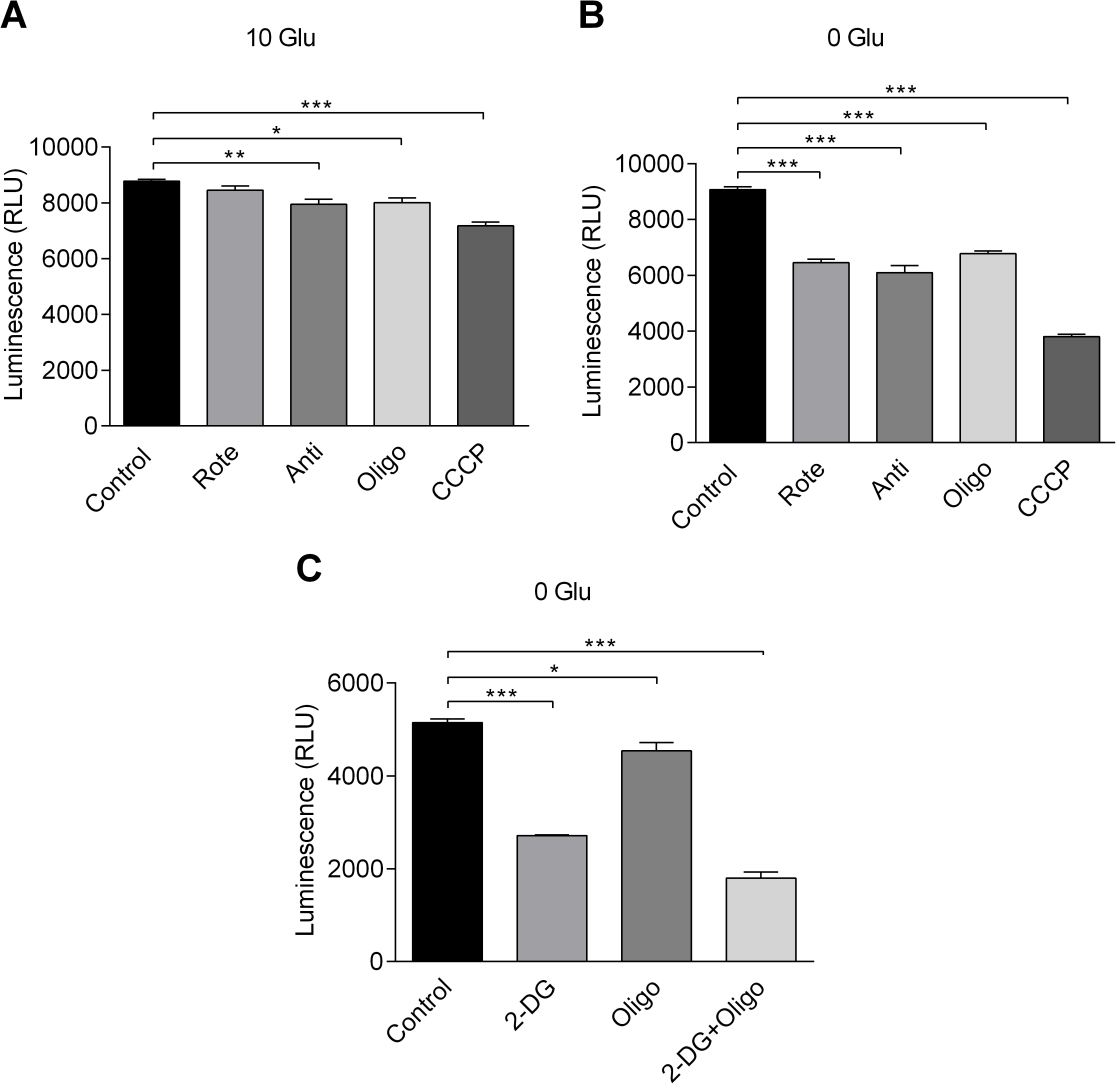
Effects of metabolic inhibitors on cellular ATP. Effect of metabolic inhibitors on cellular ATP was measured after 10 min in the presence of 10 mM glucose (A) or 0 mM glucose (B and C) (n = 4). Rote: 1 μM rotenone. Anti: 1 μM antimycin. Oligo: 6 μM oligomycin. CCCP: 1 μM CCCP. 2-DG: 5 mM 2-DG. Significant difference was assessed using ANOVA. Note that rotenone application in the presence of 10 mM glucose (panel A second from left) did not cause significant change, but all other treatments with metabolic inhibitors significantly changed luminescence reading against control values.

To further examine the effect of metabolic modulators, cytosolic ATP levels were determined using CellTiter-Glo luminescent viability assay. First, luminescent signals from known ATP concentrations and known cell numbers were measured to construct standard curves as shown in supporting information (S2 Fig). By combining the information from two curves, the intracellular ATP concentration can be estimated to be (1.48±0.03) x10^-14^ M per cell. Cell volume (V) was calculated from the equation: V = (4/3)×π×((major axis/2+minor axis/2)/2)^3^ using parameters generated from images of cells. The volume of HCASMCs was thus estimated to be 13.4±2.9 pL (n = 10). Taking V as the intracellular volume from which measured ATP originates, the ATP concentration in HCASMCs can be calculated as 1.1 mM. Next, luminescence signals were measured in the presence and absence of 10 mM glucose, and the effects of metabolic inhibitors were tested. In the presence of 10 mM glucose, application of antimycin (1 μM), oligomycin (6 μM), and CCCP (1 μM), all significantly reduced the luminescence signal, indicating a reduction in the ATP level (Fig 5A). In the absence of glucose, all three metabolic inhibitors as well as rotenone (1 μM) induced a highly significant reduction in luminescence signal (Fig 5B). Similarly, use of 2-DG in the absence of glucose induced a highly significant reduction in luminescence signal (Fig 5C). Taken together, these results suggest that metabolic inhibitors have a significant impact on the cellular ATP level.

### Effect of metabolic inhibitors on mitochondrial membrane potential

**Fig 6:**
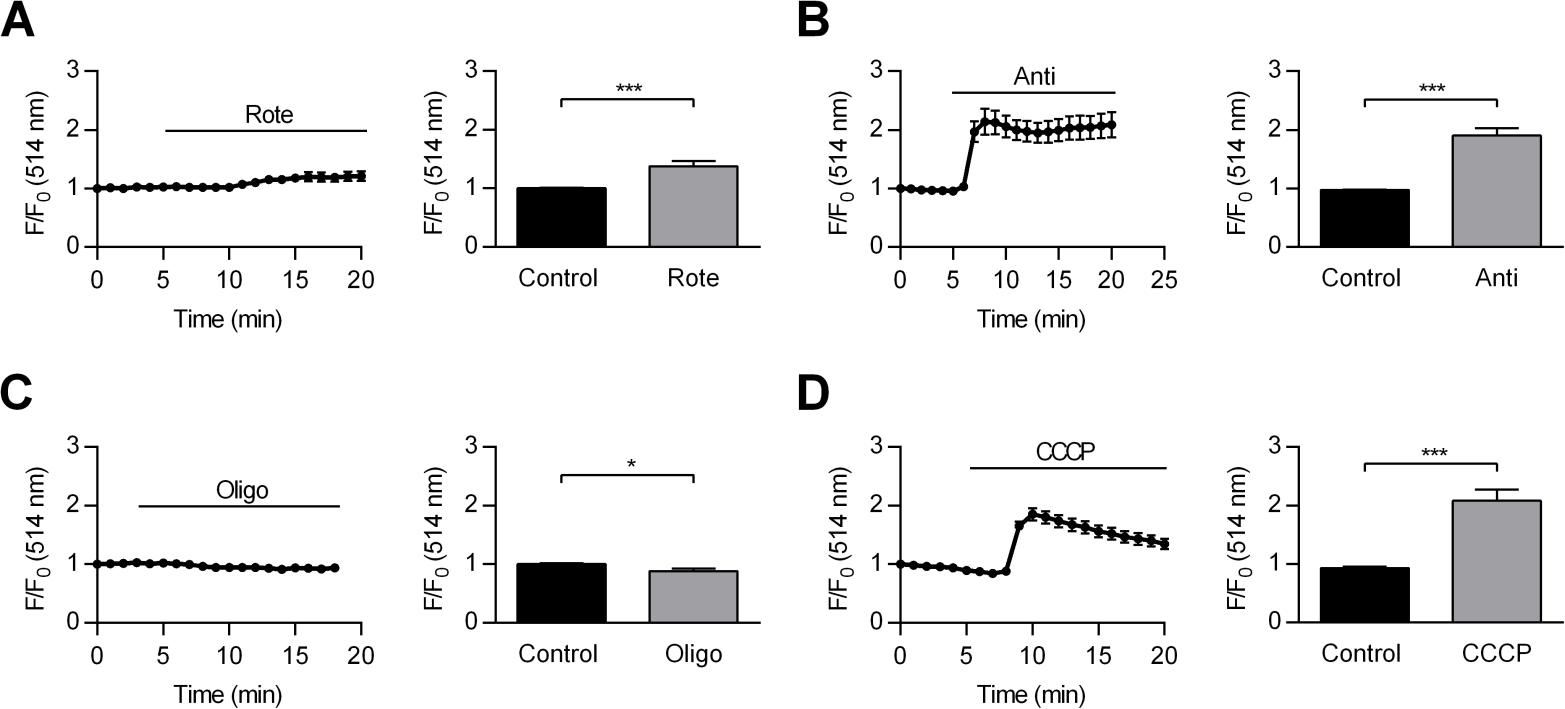
Effect of metabolic inhibitors on mitochondrial membrane potential. Left panel of each set shows change in fractional fluorescence of the cells treated with 1 μM rotenone (A), 1 μM antimycin (B), 6 μM oligomycin (C) and 1 μM CCCP (D). Right panel of each set shows mean ±SEM of fractional fluorescence before and after application of rotenone (A, p<0.001, n = 33), antimycin (B, p<0.001, n = 30), oligomycin (C, p<0.05, n = 16) and CCCP (D, p<0.001, n = 17). Statistical significance was evaluated using paired Student’s *t*-test.

**Fig 7:**
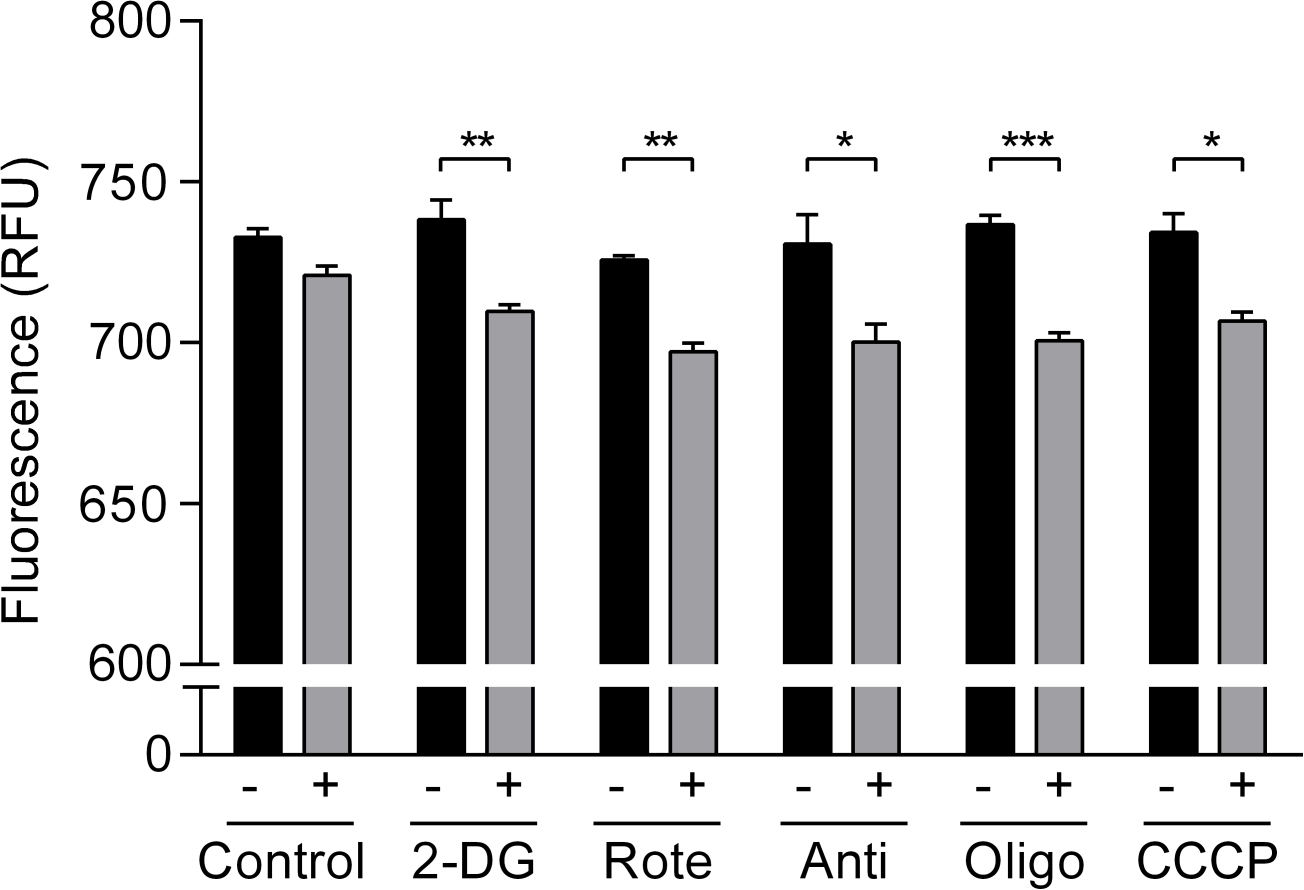
Effects of metabolic inhibitors on cellular ATP:ADP ratio. Effect of metabolic inhibitors on cellular ATP:ADP ratio measured after 15 min in the absence and presence of, left to right, DMSO (NS), 5 mM 2-DG (p<0.01), 1 μM rotenone (p<0.01), 1 μM antimycin (p<0.05), 6 μM oligomycin (p<0.001) and 1 μM CCCP (p<0.05). Statistical analysis was performed using Student’s un-paired *t* test (n = 4).

Next, we examined the effect of OXPHOS inhibitors on ψ_m_. With the exception of oligomycin which blocks ATP synthase, metabolic inhibitors reduce ATP levels by interfering with the ETC and causing ψ_m_ depolarization. Mitochondrial membrane potential was measured in single cells using the potentiometric dye Rhodamine123. Rhodamine123 signal was stable over the duration of experiments (time matched control, data not shown) and expressed as fractional fluorescence (F/F_0_) where F_0_ is the baseline measurement at the start of experiments. Fig 6A left panel shows an increase in fractional fluorescence following application of rotenone. Accumulation of rhodamine123 to mitochondria is driven by very negative ψ_m_, leading to dye quenching [17, 21]. With mitochondria depolarization, some dye leaves mitochondria and is de-quenched, increasing the signal intensity. Fig 6A right panel shows the mean ± SEM of fractional fluorescence just before and 15 min after rotenone application. Rhodamine123 signal increased by 37.80±8.39% after application of 1 μM rotenone (Fig 6A right panel, p<0.001), suggesting ψ_m_ depolarization. Likewise, 1 μM antimycin caused an increase in fractional fluorescence (Fig 6B, left panel) by 90.18±13.05% (Fig 6B, right panel, p<0.001). However, application of 6 μM oligomycin caused a decrease in fractional fluorescence (Fig 7C, left panel) by 11.8±4.44% (Fig 6C, right panel, p<0.05). Finally, application of 1 μM CCCP caused a bi-phasic increase in fractional fluorescence (Fig 6D, left panel) by 108.29±18.63% measured at the peak (Fig 6D, right panel, p<0.001). Example images of these experiments are shown in supporting information (S3 Fig).

### Effects of metabolic inhibitors on cellular ATP:ADP ratio

As described earlier, intracellular ATP also serves as an intracellular signaling molecule, and its targets may include K_ATP_ channels. In the context of the coronary artery, activating K_ATP_ channels during increased metabolic demand may be important in matching the supply of blood. For example, genetically modified mice lacking K_ATP_ subunits (Kir6.1^-/-^ or SUR2^-/-^) have exhibited coronary vasospasm that resembled human Prinzmetal angina [11, 12]. One possible link between K_ATP_ channel activation and increased metabolic demand is the reduction in ATP:ADP ratio [11, 12]. Thus, the effect of metabolic modulators on cellular ATP:ADP ratio was evaluated using FUGW-PercevalHR [14]. Infection of HCASMCs with 3^rd^ generation lentivirus incorporating FUGW-PercevalHR gave an expression efficiency of near 100% allowing the signal to be measured using a multi-well plate format. Inhibition of glycolysis or OXPHOS using metabolic inhibitors for 15 min caused a significant reduction in the FUGW-PercevalHR signals expressed as relative fluorescent unit (RFU) (Fig 7) suggesting relative decrease in ATP and increase in ADP level.

## Discussion

We have presented a cellular bioenergetic analysis of coronary arterial smooth muscle cells determined using the Seahorse technique. Preliminary experiments confirmed virtually all cells were of smooth muscle origin, and neither expression of smooth muscle specific proteins nor parameters of cell morphology changed over passages (data not shown). Thus, the shift in bioenergetic phenotype seen during cell culture (Fig 4) can be attributed to the aging of the cell population, rather than increase in the proportion of non-smooth muscle cells. With the increase of the HCASMC passage number, the proportion of both OCR linked to ATP synthase and respiratory reserve capacity decreased (Fig 4). The latter is thought to be an important indicator of healthy mitochondria, providing protection against possible metabolic insult. The high detection sensitivity of the technique also permitted isolation of non-mitochondrial OCR and non-glycolytic ECAR thereby generating the indices of true basal OCR and ECAR (Fig 1). These parameters could not have been measured by more traditional methods and may be important in cellular bioenergetics. For example, it has been reported that redox cycling agents significantly increased non-mitochondrial OCR in bovine aortic endothelial cells [9]. It is also interesting to note that unexpectedly high non-mitochondrial OCR was reported for rat hepatocytes and cardiomyocytes [1]. Together with the result presented here, the traditional view that over 95% of oxygen consumption is due to mitochondrial respiration may require re-evaluation [7].

The estimation of intracellular ATP production at rest in HCASMCs showed that HCASMCs produce 54.5% of ATP through OXPHOS with remaining 45.5% due to glycolysis (Fig 2). Note that functional compartmentalization of ATP production/consumption has been postulated where aerobic glycolysis was tightly linked to Na^+^ pump activities in the plasma membrane while artery contraction specifically enhanced OXPHOS [5]. Nevertheless, the proportion of glycolysis seems somewhat high but this does not necessarily mean HCASMCs are glycolytic cells. Seahorse experiments were carried out using cells not experiencing load/stretch. However, artery smooth muscle cells are somewhat contracted in order to maintain vascular tone *in vivo*, and this requires the muscle cells to be working. With increases in metabolism, cells require higher amounts of ATP that may be produced via OXPHOS *in vivo*. OCR due to ATP production was 3.27 pM/min/μg protein while respiratory reserve was 8.27 pM/min/μg protein, indicating the respiratory reserve capacity was 250% of the ATP synthase-linked O_2_ consumption at rest (Fig 1A). On the other hand, ECAR due to glycolysis was 7.86 mpH/min/μg protein at rest while glycolytic reserve was 1.87 mpH/min/μg protein, suggesting glycolysis can increase only by a quarter of the resting level (Fig 1C). Furthermore, non-glycolytic acidification, likely to be due to respiratory acidification where CO_2_ hydrates to form H_2_CO_3_ which dissociates to HCO_3_ and H^+^, was 2.45 mpH/min/μg protein, equating 31% of glycolysis ECAR (Fig 1C). Our results show that HCASMCs have healthy mitochondria with high reserve capacity, conferring resilience under stress, and this may have an important implication regarding protection of the heart. It has been suggested that growth of coronary collaterals, thought to be crucial during compromised coronary blood flow, may rely on arterial mitochondrial function [22]. Arteriogenesis such as coronary collateral growth requires phenotypic switching of cells, and it has been suggested that the energetic capacity and reserves may play a key part during vascular growth [22]. Although more studies are required, the notion that bioenergetic components are involved in the adaptive process is intriguing.

Sequential application of pharmacological agents to dissect defined components of OCR and ECAR relies on the specificity of the metabolic modulators. Clearly, these chemicals also have off-target effects, but if the order of application and deployed concentration are chosen with care, such experiments still provide valuable insight to cellular bioenergetics [9]. One crucial caveat in the Seahorse experiments is, however, while oligomycin concentration must be high enough to fully inhibit ATP synthase, a supramaximal dose will continue to increase OCR [9]. This OCR component is ATP synthase independent and could be due to increased proton leak related oxygen consumption [23]. Furthermore, the stimulation of OCR with FCCP is bell-shaped where initial stimulation is followed by inhibition [9]. Simply adopting a concentration determined using different cell types is strongly discouraged and careful combined titration of oligomycin and FCCP is necessary for each cell type subjected to Seahorse technique [1, 9]. Thus, oligomycin and FCCP at a concentration of 1.0 μM and 0.75 μM were chosen for HCASMCs based on the results shown in supporting information S1 Fig.

When bioenergetic phenotype shift were examined, rotenone, antimycin and oligomycin caused the cells to become more glycolytic (Fig 3C, 3D & 3E), indicating that glycolysis allows compensatory production of ATP when OXPHOS is inhibited. Also, extracellular glucose blunted reduction in ATP level caused by metabolic inhibitors (Fig 5). Moreover, application of vasoconstrictors, PDGF-BB (20 ng/ml) and PGF2α (10 μM), did not cause any change in ATP level in the presence of 10 mM glucose although these drugs reliably triggered/enhanced Ca^2+^ oscillations in HCASMCs (data not shown). Combined with high respiratory reserve capacity, HCASMCs appear to be able to deal with acute metabolic demand with well-maintained ATP levels [24].

The intracellular ATP concentration of HCASMCs was calculated to be 1.1 mM from the results of CellTiter-Glo luminescent viability assay and estimated cell volume. However, the cell volume calculation is likely to be an overestimation of cytosolic volume due to the presence of intracellular organelles. Thus, the value of 1.1 mM is at the lower end of the estimate, and the true value in HCASMCs may be considerably higher than this. In future studies, it will be interesting to perform Seahorse stress tests in the presence of vasoconstrictors. Although the total ATP level did not change with the application of contractile agents in these cells, contribution of OXPHOS may still increase at the expense of glycolysis when HCASMCs are made to work due to the Pasteur effect [2].

In terms of change in ψ_m_, all metabolic inhibitors with the exception of oligomycin caused a significant increase in rhodamine123 fractional fluorescence, suggesting mitochondrial depolarization (Fig 6). When ATP synthase is operating in forward mode, production of ATP is accompanied by proton re-entry and thus a small ψ_m_ depolarization. The by-product of ATP synthase inhibition by oligomycin, therefore, is an inhibition of proton re-entry, and this will lead to a small ψ_m_ hyperpolarization [21]. Application of CCCP and collapse of ψ_m_ on the other hand, drive ATP synthase into reverse mode in a vain attempt to restore ψ_m_. The biphasic increase in rhodamine123 fractional fluorescence may thus reflect a partial recovery of ψ_m_ at the cost of ATP consumption (Fig 6D). Note that, in the presence of glucose, application of rotenone did not cause a significant change in ATP level (Fig 5A), and this may be explained by a relatively modest ψ_m_ depolarization caused by rotenone (Fig 6A). Supporting information S3 Fig shows example images of rhodamine123 loaded HCASMCs before and after application of metabolic inhibitors.

Though it is useful to measure cytosolic ATP levels, the absolute value might vary among different cell types and even within the same cell type. More importantly, some metabolically activated effectors including K_ATP_ channels are known to be regulated not only by ATP but also ADP [25]. Such dual regulation presumably is more effective as finer tuning can be achieved. Thus, we determined changes in ATP:ADP using biosensors. We initially sought to transiently transfect HCASMCs with PercevalHR. However, the rate of transfection was very low. Thus, HCASMCs expressing FUGW-PercevalHR transduced by a 3^rd^ generation lentivirus were generated. Representative images of biosensor expressing HCASMCs are shown in supporting information S4 Fig. As virtually all HCASMCs expressed FUGW-PercevalHR, it was possible to conduct microplate experiments. As shown in Fig 7, OXPHOS and glycolysis inhibitors including rotenone caused significant change in FUGW-PercevalHR RFU. PercevalHR, and its predecessor Perceval, were created to detect change in intracellular ATP:ADP ratio [13, 14], but it has been reported that the biosensor is insensitive to ADP and only detects ATP level [15]. Our PercevalHR *in vivo* calibration using permeablized HCASMCs indicated that PercevalHR signal does change with ADP level at a given concentration of ATP (data not shown). Thus, PerveralHR fluorescence is high when ATP is high and ADP is low, and decrease in PercevalHR signal implies decrease in ATP and increase in ADP level. Note that application of complex I inhibitor, rotenone, significantly reduced only PercevalHR signal (Fig 7) but not ATP level in the presence of extracellular glucose (Fig 5). This suggests that ATP:ADP ratio may be a useful way to translate subtler change in cellular metabolic status to physiological response.

The present work provides insight into the bioenergetics of HCASMCs gained from the Seahorse technology, ATP luminescence, ψ_m_ and FUGW-PercevalHR experiments. The ultimate goal is to map out changes in bioenergetic phenotype that may be closely linked with health and disease in cardiovascular systems. The development of vascular diseases ranging from atherosclerosis to neo-intimal hyperplasia is thought to be associated with proliferation and migration of smooth muscle cells [6]. Triggers and signal transduction cascades may vary, but stimulation of cell growth and change in metabolic phenotype are likely to play a pivotal role shared among vascular diseases. It follows that, even before more conventional parameters such as ATP levels and mitochondrial function are affected, more subtle changes in cellular bioenergetics might have already started the pathological process. Similarly, it will be interesting to examine how the age of donor may influence the cellular bioenergetics in the future. Literally, coronary arteries supply life blood to the heart, and well regulated coronary blood flow can and does mitigate potential damage to cardiac function. It has been over 30 years since Paul measured oxygen consumption and lactate production from intact porcine coronary arteries, proposing the compartmentalization of ATP production and consumption [5]. Recent development in methods including Seahorse and PercevalHR provide unprecedented opportunities to uncover bioenergetics of living cells. From the relatively limited number of publications so far [eg, 1, 3, 6, 7, 9], it seems that many conventional views of cellular bioenergetics need to be re-evaluated. So long as carefully planned and accurately executed, studies such as present paper will provide useful information in understanding of bioenergetics of vascular smooth muscle cells.

## Conclusions

The Seahorse technique revealed a detailed breakdown of cellular bioenergetics of proliferating HCASMCs. HCASMCs rely equally on OXPHOS and glycolysis at rest and have high respiratory reserve capacity and low glycolysis reserve capacity. Aging cells have lower resting OXPHOS with reduced reserve capacity. Intracellular adenylate nucleotides may serve as key signaling molecules during physiological and pathological events that affect cellular metabolism. Understanding of cellular bioenergetics of artery smooth muscle cells is an integral part of cardiovascular systems in health and disease.

## Supporting information

S1 Fig Optimization of FCCP and oligomycin concentrations. OCR determined with different FCCP concentrations is plotted for a given oligomycin concentration, 0.5, 1.0, 1.5 or 2.0 μM. FCCP shows a bell-shaped concentration response, and a concentration of 0.75 μM, shown in black triangles, was optimal to cause a maximal effect (n = 4). Metabolic inhibitors were added sequentially as shown in Fig 1A. These data were re-plotted for given FCCP concentrations to determine optimal oligomycin dose, and concentration of 1.0 μM was chosen.
(TIF)

S2 Fig CellTiter-Glo luminescent viability assay standard curves. ATP standard curve was constructed as a linear fit of luminescence outputs either as a function of ATP concentration (*A*, n = 6) or cell number (*B*, n = 6).
(TIF)

S3 Fig Effect of metabolic inhibitors on rhodamine123 signals. Images of HCASMCs before (left panels) and after (right panels) application of 1 μM rotenone, 1 μM antimycin, 6 μM oligomycin, and 1 μM CCCP. Scale bar: 100 μm.
(TIF)

S4 Fig Images of HCASMCs expressing PercevalHR and FUGW-PercevalHR. HCASMCs were either transfected with reagent (left panels) or infected with lentivirus to express biosensors. From top to bottom, bright field, PercevalHR/FUGW-PercevalHR expressing cells and overlay. Scale bar: 400 μm.
(TIF)
