# Supplementary figures and images for: Bioenergetic profile of human coronary artery smooth muscle cells and effect of metabolic intervention
Source: PLoS One. 2017 May 19;12(5):e0177951. doi: 10.1371/journal.pone.0177951 (PMC5438125; doi:10.1371/journal.pone.0177951)

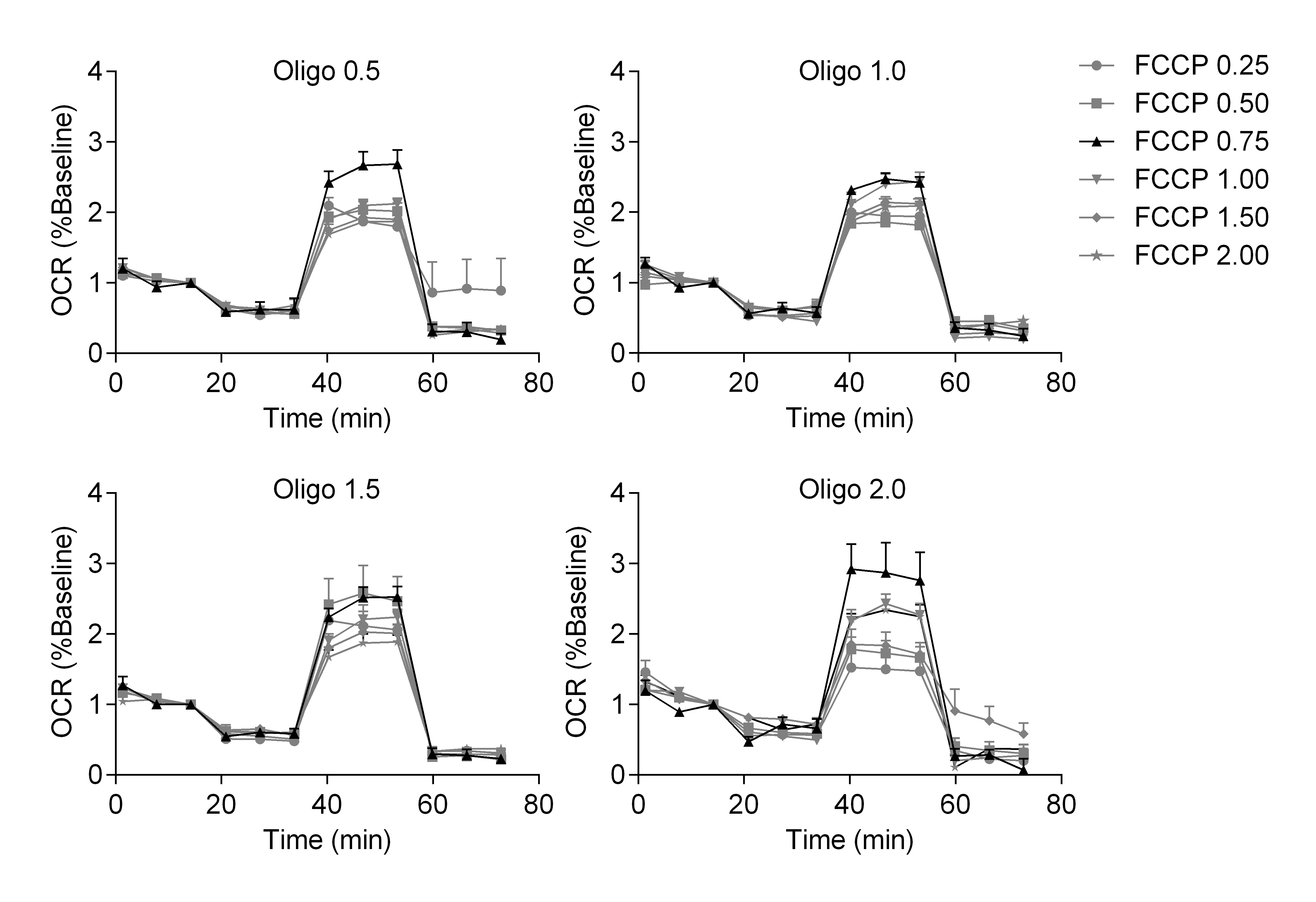

Supplement: S1 Fig — OCR determined with different FCCP concentrations is plotted for a given oligomycin concentration, 0.5, 1.0, 1.5 or 2.0 μM. FCCP shows a bell-shaped concentration response, and a concentration of 0.75 μM, shown in black triangles, was optimal to cause a maximal effect (n = 4). Metabolic inhibitors were added sequentially as shown in Fig 1A. These data were re-plotted for given FCCP concentrations to determine optimal oligomycin dose, and concentration of 1.0 μM was chosen. (TIF) [file pone.0177951.s001.tif]

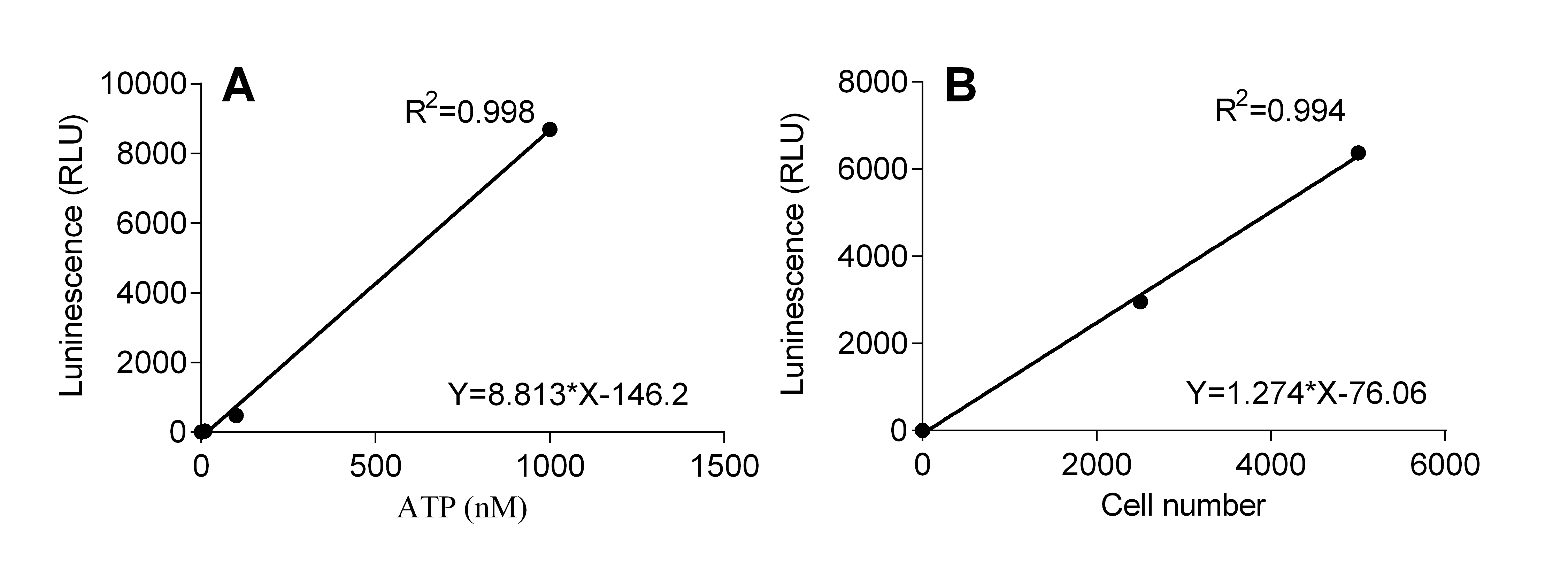

Supplement: S2 Fig — ATP standard curve was constructed as a linear fit of luminescence outputs either as a function of ATP concentration (A, n = 6) or cell number (B, n = 6). (TIF) [file pone.0177951.s002.tif]

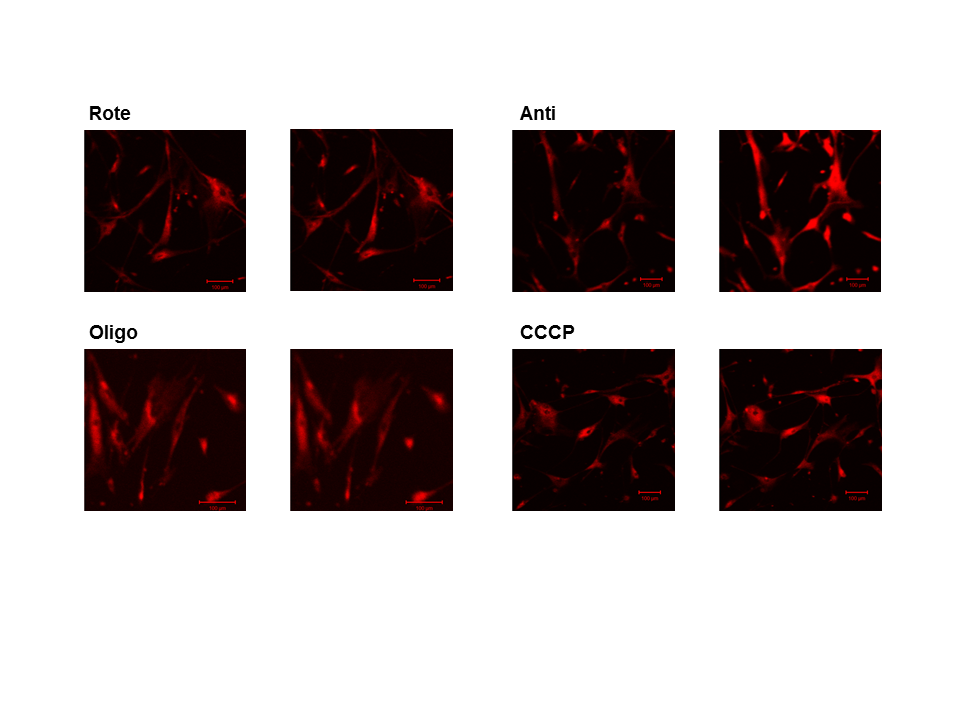

Supplement: S3 Fig — Images of HCASMCs before (left panels) and after (right panels) application of 1 μM rotenone, 1 μM antimycin, 6 μM oligomycin, and 1 μM CCCP. Scale bar: 100 μm. (TIF) [file pone.0177951.s003.TIF]

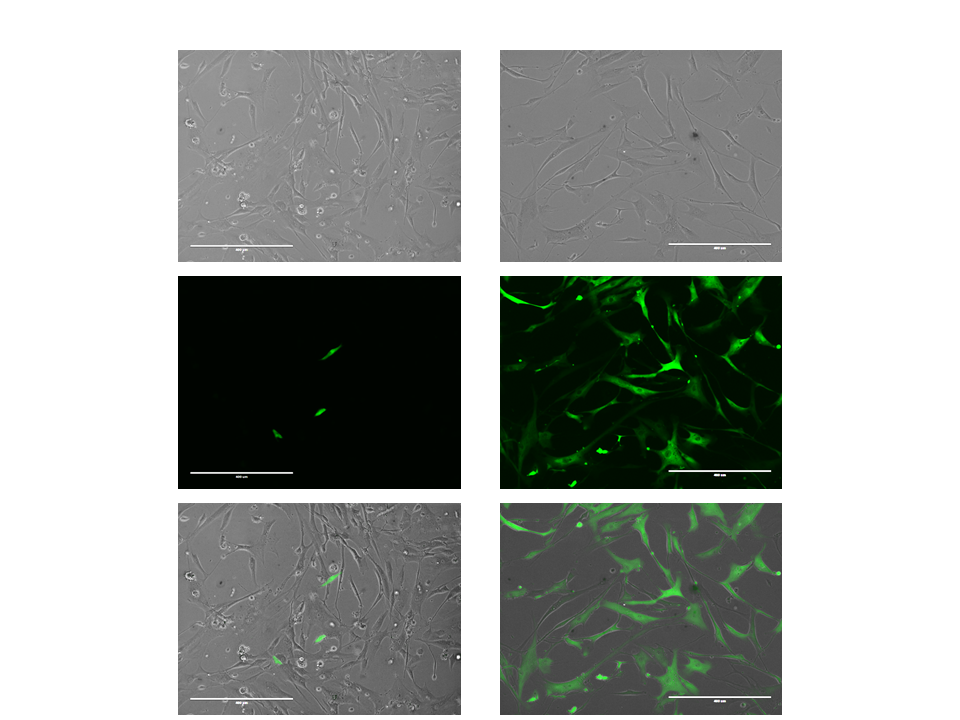

Supplement: S4 Fig — HCASMCs were either transfected with reagent (left panels) or infected with lentivirus to express biosensors. From top to bottom, bright field, PercevalHR/FUGW-PercevalHR expressing cells and overlay. Scale bar: 400 μm. (TIF) [file pone.0177951.s004.TIF]
